# Trends of Early *Helicobacter pylori*-Uninfected Gastric Cancer in an Aging Regional Area

**DOI:** 10.3390/jcm13061827

**Published:** 2024-03-21

**Authors:** Hidehito Maeda, Fumisato Sasaki, Takayuki Ooi, Shohei Uehara, Hiroki Yano, Yoichi Sameshima, Yoshio Fukuda, Yuichiro Nasu, Yusuke Fujino, Koichiro Shigeta, Hiroshi Fujita, Akihito Tanaka, Shuji Kanmura, Akio Ido

**Affiliations:** 1 Digestive and Lifestyle Diseases, Kagoshima University Graduate School of Medical and Dental Sciences, Kagoshima 890-8520, Japan; hidehitomaeda@gmail.com (H.M.);; 2 Division of Gastroenterology, Kagoshima Prefectural Oshima Hospital, Amami 894-0015, Japan; 3 Division of Gastroenterology, Kagoshima Kouseiren Hospital, Kagoshima 890-0062, Japan; 4 Division of Gastroenterology, Kagoshima City Hospital, Kagoshima 890-8760, Japan; 5 Division of Gastroenterology, Saiseikai Sendai Hospital, Satsumasendai 895-0074, Japan; 6 Division of Gastroenterology, Kirishima Medical Center, Kirishima 899-5112, Japan; 7 Division of Gastroenterology, Izumi General Medical Center, Izumi 899-0131, Japan

**Keywords:** *Helicobacter pylori*-uninfected gastric cancer, aging regional area, early gastric cancer, endoscopic submucosal resection

## Abstract

**Background/Objectives**: We aimed to determine the trends over time and current status of early *Helicobacter pylori*-uninfected gastric cancer (HpUIGC) treatment in a region with an aging population. **Methods**: This retrospective, multi-center observational study was conducted at seven major general hospitals in Kagoshima Prefecture. From January 2009 to July 2022, 2091 patients who received endoscopic resection (ER) for early gastric cancer (EGC) were retrospectively enrolled, of which 35 were identified as early HpUIGC cases. **Results**: The number of ERs for EGC demonstrated a significant increasing trend from 2010 to 2021 (*p* = 0.01 for trend). Furthermore, the 12-year period from 2010 to 2021 was divided into an early and late phase every 6 years. In the early phase, there were 5 cases (0.7%) of early HpUIGC, while in the late phase, there were 25 cases (2.1%), indicating a significant increase in the proportion of ERs for early HpUIGC cases in the late phase (*p* = 0.02). **Conclusions**: The proportion of ERs for early HpUIGC, which are more common in relatively young patients, may be increasing as a proportion of all ERs for GC, even in areas of Japan with an aging population.

## 1. Introduction

*Helicobacter pylori* infection is a confirmed carcinogenic factor that triggers atrophy and intestinal metaplasia in the gastric mucosa, increasing the likelihood of developing gastric cancer (GC) [1]. In a prospective study, Fukase et al. demonstrated that eradication of *H. pylori* reduced the incidence of metachronous GC in one-third of patients [2]. As a result of these research outcomes, the eradication therapy for *H. pylori* has been included in Japanese insurance coverage since 2013 and was extensively administered to patients infected with *H. pylori*. Owing to the combination of widespread eradication therapy and improvements in living conditions and sanitation, there has been a decline in the rate of *H. pylori* infection in recent years, especially among young people [3]. Therefore, age-adjusted incidence rates have shown a downward trend; however, GC is detected more frequently in aging populations, and the crude incidence rate of GC continues to increase owing to the aging population [4]. Under such circumstances, the number of GC cases detected at an early stage has been increasing in recent years owing to improved endoscopic diagnostic techniques and screening [4]. Furthermore, endoscopic submucosal dissection (ESD) for early-stage GC has been established as a standard treatment and is widely used [5]. Owing to early detection and improved treatment outcomes, the age-adjusted mortality rate of GC in Japan has shown a pronounced downward trend [4]. Early detection of GC and a decreasing trend in *H. pylori* infection are expected to lead to a further reduction in GC-related mortality in Japan. 

Recent reports have, however, described the occurrence of GC in relatively young patients in their 50s–60s without *H. pylori* infection, a condition referred to as *H. pylori*-uninfected GC (HpUIGC) [6,7,8,9]. HpUIGC accounts for 0.42–5.4% of all GC cases and, although rare, it is likely to increase relatively in the future as the number of *H. pylori*-infected GC (HpGC) cases declines [8,9,10,11,12].

Kagoshima Prefecture, where this study was conducted, is located in southern Japan. The aging of the population, which has become a problem in Japan, is evident in the Kagoshima Prefecture, where the population of those aged 65 years and older is extremely high at 32.5% compared to the national average of 28.6% [13].

There have been no reports on the number of patients with early-stage HpUIGC in an area with an aging population, such as Kagoshima Prefecture. It is crucial for gastroenterologists to comprehend the current status of early HpUIGC. This is also important for future GC treatment in major cities, as the population is expected to continue to age. It may also provide useful information for GC screening to decrease GC mortality in Japan, Korea, and other countries. In this study, we aimed to ascertain the trends over time and the current status of early HpUIGC treatment in a region with an aging population.

## 2. Materials and Methods

### 2.1. Patients

This retrospective, multi-center observational study was conducted by the Kagoshima ESD Group (Kagoshima University Hospital, Kagoshima Kouseiren Hospital, Kagoshima City Hospital, Saiseikai Sendai Hospital, Kirishima Medical Center, Izumi General Medical Center, and Kagoshima Prefectural Oshima Hospital) created by seven major general hospitals in Kagoshima Prefecture. From January 2009 to July 2022, 2091 patients who received endoscopic resection (ER) for early GC (EGC) were retrospectively enrolled, of which 35 cases were identified as early HpUIGC. Esophagogastric junction (EGJ) cancers include some cases of HpUIGC; however, because distinguishing between some cases of HpUIGC and Barrett’s esophageal cancer can be challenging, we excluded cases of EGJ cancers in the present study.

### 2.2. Determination of H. pylori-Uninfected Status

Referring to previous reports, *H. pylori*-uninfected cases were defined based on the following criteria: (1) no history of *H. pylori* eradication; (2) no endoscopic evidence of gastric atrophy and testing negative on any one *H. pylori* infection assay (13C-urea breath test, serum immunoglobulin G antibodies against *H. pylori*, or *H. pylori* stool antigen test); and (3) testing negative on at least two *H. pylori* infection assays; 1 + 2 or 3 were defined as *H. pylori*-uninfected [6,8].

### 2.3. Date Collection

The primary endpoint was the change over time in the proportion of early HpUIGC cases to the total number of ERs for EGC cases. First, we examined the number of ERs for EGC cases in Japan from 2014 to 2021 using published data from the National Database (NDB) [14]. Next, we examined the number of ERs for EGC in the Kagoshima ESD Group from 2010 to 2021. Furthermore, the 12-year period from 2010 to 2021 was divided into an early and late phase every 6 years, and the proportion of early HpUIGC cases to the total number of ERs for EGC cases during the same period was compared. 

The secondary endpoint was the clinical characteristics of early HpUIGC cases, including age, sex, opportunity to detect lesions, resection modality, tumor location, macroscopic type, lesion size, histological type, lesion depth, and treatment outcomes such as en bloc resection rate, horizontal margin positive rate, vertical margin positive rate, curability of ER, and complications rate. 

Tumor location was determined using the Japanese Classification of Gastric Carcinoma (JCGC) classification, categorizing it into the upper, middle, and lower thirds of the stomach [15]. En bloc resection was defined as resection in a single piece as opposed to resection of multiple pieces. Curability of ER was evaluated by eCura A, eCura B, and eCura C using the Japanese GC treatment guidelines [16]. Endoscopic diagnosis and/or the identification of free air on a plain radiograph or computed tomography (CT) immediately post-resection determined perforation, and delayed perforation was defined as the sudden emergence of abdominal pain and signs and symptoms of peritoneal irritation accompanied by the detection of free air on chest and abdominal radiography or abdominal CT in a patient who showed no evidence of perforation during ER or free air immediately post-ER [17,18]. Postoperative bleeding was defined as the occurrence of evident hematemesis or melena, or a decrease of 2 g/dL in hemoglobin levels along with changes in vital signs after ER, in which bleeding from the mucosal defect or blood in the stomach was confirmed by emergency endoscopy [19]. Additionally, the 3-year survival rate was examined in 12 patients with early HpUIGC who could be followed for 3 years. This study was conducted in accordance with the tenets of the Declaration of Helsinki and was approved by the Institutional Review Board of Kagoshima University (180332).

### 2.4. Statistical Analysis

The Jonckheere–Terpstra test was used for trend analysis. The change over time in the proportion of HpUIGC in the ER of EGCs was analyzed using a linear regression model. Differences between the two groups were assessed using a Chi-square test. *p*-values < 0.05 were considered significant. All statistical analyses were performed with the Statistical Package for the Social Sciences software version 22 (IBM Corp.; Armonk, NY, USA). 

## 3. Results

### 3.1. Characteristics of the Patients and Lesions of Early HpUIGC and Treatment Outcome

**Figure 1:**
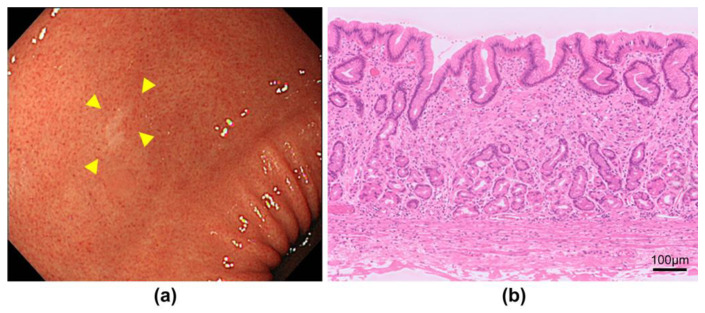
Endoscopic and pathological findings of signet-ring cell carcinoma. (**a**) White light image shows a small fading 0-IIc lesion (yellow arrows); (**b**) Pathology shows signet ring-cell carcinoma in the middle layer of the mucosa.

**Figure 2:**
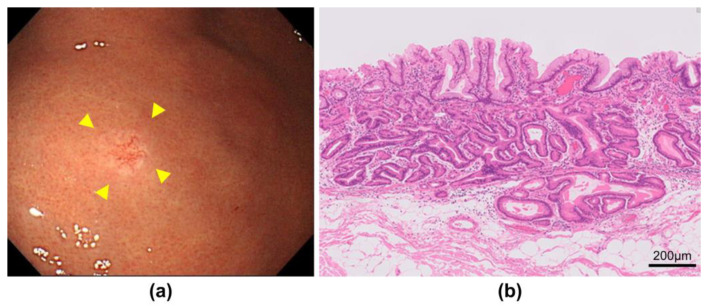
Endoscopic and pathological findings of gastric adenocarcinoma of fundic-gland-type. (**a**) White light image shows a small 0-IIa lesion with dilated vessels on the surface of the lesion (yellow arrows); (**b**) Pathology shows gastric adenocarcinoma of fundic-gland-type invading the submucosa.

**Figure 3:**
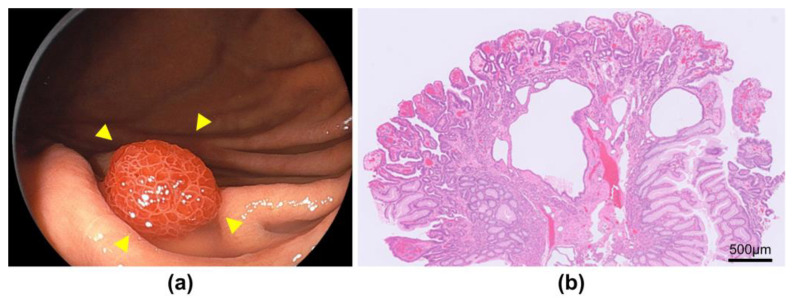
Endoscopic and pathological findings of foveolar-type adenocarcinoma with a raspberry-shaped appearance. (**a**) White light image shows a small reddish 0-I lesion (yellow arrows); (**b**) Pathology shows well-differentiated adenocarcinoma that mimicked foveolar epithelium in the superficial layer.

Of the 2091 patients enrolled, 35 patients (1.7%) had early HpUIGC. The mean age of the patients was 55.7 years, and 75% of patients with HpUIGC were male. Almost all (97.1%) of the opportunities for detecting HpUIGC were through checkups and regular esophagogastroduodenoscopy. The resection modality for HpUIGC was ESD in 31 cases (88.6%). The median lesion size of HpUIGC was 5 mm. The most common histological type of HpUIGC was signet-ring cell carcinoma (SRCC) (Figure 1) at 40.0%, and gastric adenocarcinoma of the fundic-gland-type (GA-FG) (Figure 2) at 31.4%, accounting for approximately 70% of all cases. Foveolar-type adenocarcinoma (GA-FV) with a raspberry-shaped appearance (GA-FV-RS) (Figure 3) accounted for 17.1%. The lesion depth of HpUIGC was the mucosa (M) in 23 cases (65.7%) and submucosa 1 (SM1) in 10 cases (28.6%). There was only one case each of lymphatic and vascular invasion (Table 1).

The en bloc resection rate of HpUIGC was 100%, and the positive rate of horizontal margin was 0%; however, a positive vertical margin was observed in one case. Evaluation of curability showed three cases (8.6%) of eCura C in HpUIGC. Regarding complications, intraoperative perforation was observed in one case (Table 2). 

### 3.2. Clinical and Pathological Characteristics

**Figure 4:**
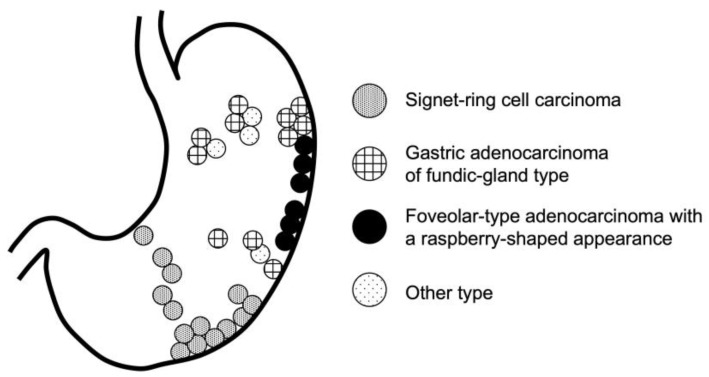
Location of the early *Helicobacter pylori*-uninfected gastric cancer in this study. Signet-ring cell carcinomas were found in the border region between the fundic and pyloric glands of the stomach. Among gastric adenocarcinoma of fundic-gland-type, eight cases (72.7%) were found in the upper area. All six cases of foveolar-type adenocarcinoma with a raspberry-shaped appearance were found in the greater curvature region.

We showed the location of early HpUIGC in this study (Figure 4). SRCC cases were found in the border region between the fundic and pyloric glands of the stomach. Among GA-FG cases, eight cases (72.7%) were found in the upper area. All six cases of GA-FV-RS were found in the greater curvature region. Regarding the macroscopic type, there were no 0-I or 0-IIa lesions in the SRCC, and eight cases (72.7%) showed 0-IIa lesions in the GA-FG. Furthermore, regarding GA-FV-RS, five cases (83.3%) had 0-I lesions. In terms of lesion size, the median for SRCC, GA-FG, and GA-FV-RS was less than 10 mm. The depth of GA-FG was SM1 in nine cases (81.8%) (Table 3).

### 3.3. The 3-Year Survival Rate after Endoscopic Resection

Twelve out of the 36 early HpUIGC cases were followed for more than 3 years. Among them, six cases were classified as eCura A, three as eCura B, and three as eCura C2, with additional surgery performed in all eCura C2 cases. The 3-year survival rate was 100%, and no cases of metastatic recurrence were observed.

### 3.4. Change in the Proportion of Early HpUIGC to the Total Number of ERs for EGC

**Figure 5:**
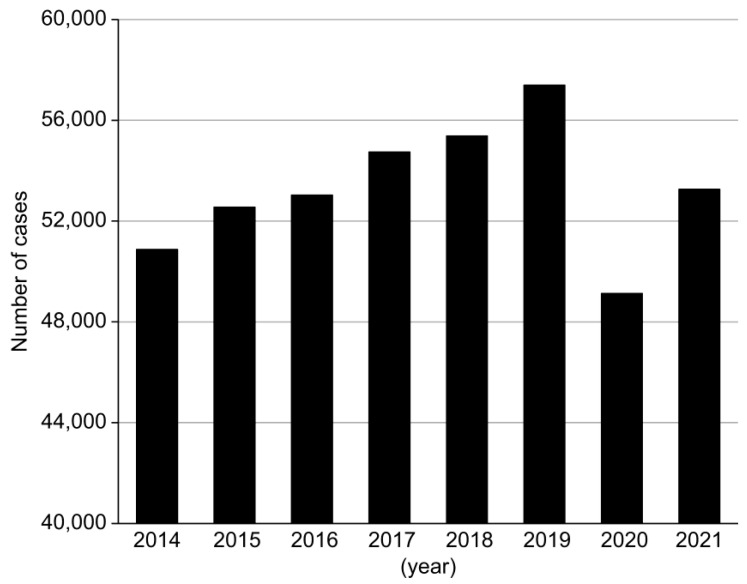
Number of endoscopic resections for early gastric cancer in the national database. The number of endoscopic resections for early gastric cancer did not show a significantly increasing trend (*p* = 0.22 for trend).

**Figure 6:**
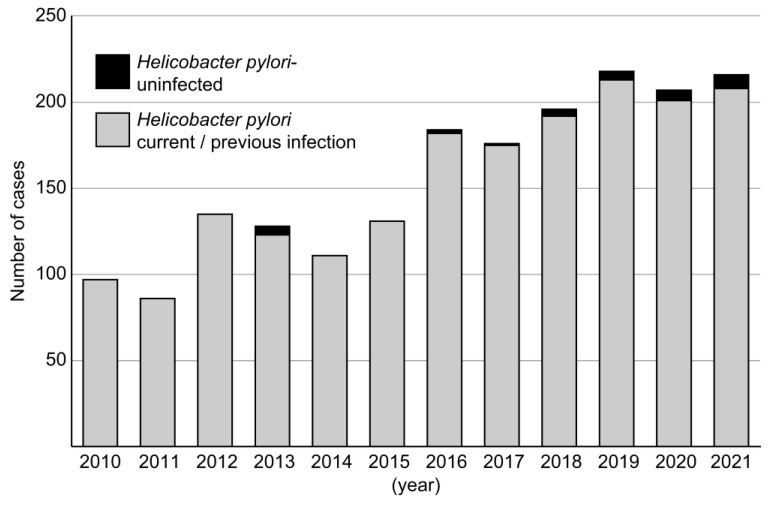
Number of endoscopic resections for early gastric cancer and proportion of early *Helicobacter pylori*-uninfected gastric cancer. The number of endoscopic resections for early gastric cancer in this study demonstrated a significant increasing trend from 2010 to 2021 (*p* = 0.01 for trend). The proportion of *H. pylori*-uninfected gastric cancer in the endoscopic resections of all early gastric cancers increased significantly in the late phase compared to that in the early phase (*p* = 0.02 for trend).

In the NDB from 2014 to 2021, the number of ERs for EGC did not show a statistically significant increasing trend (*p* = 0.22 for trend) (Figure 5). In contrast, the number of ERs for EGC in this study demonstrated a significant increasing trend from 2010 to 2021 (*p* = 0.01 for trend) (Figure 6). Regarding the proportion of HpUIGC in ERs for EGC, the linear regression model revealed *p* = 0.034 from the analysis-of-variance table; the regression coefficient was also significant at *p* = 0.034. In the early phase, the total number of ERs for EGC was 688 cases, while in the late phase, the number increased 1.7-fold to 1197 cases. In the early phase, there were five cases (0.7%) of early HpUIGC, while in the late phase, there were 25 cases (2.1%), indicating a significant increase in the proportion of ERs for early HpUIGC cases in the late phase (*p* = 0.02) (Table 4).

## 4. Discussion

The number of ERs for EGC did not exhibit a significant increase in Japan. However, in Kagoshima Prefecture where the population is aging, there was a significant increasing trend in the number of ERs for EGC. Furthermore, even in rural areas with aging populations where this study was conducted, early HpUIGC cases accounted for 1.7% of all EGC cases. These results are consistent with those in previous reports [8,9,10,11,12]. Additionally, there was a significant increase in the proportion of early HpUIGC cases during the later phase. Furthermore, since the efficacy of endoscopic screening was previously demonstrated in Japan, a population-based GC screening program was initiated in 2016, necessitating active efforts for accuracy control [20]. Therefore, a comprehensive understanding of the characteristics of early HpUIGC is extremely important for endoscopists in charge of health checkups, even in regional areas with an aging population, who must possess sufficient knowledge of the disease. 

Akazawa et al. reported that patients with HpUIGC were significantly younger than those with HpGC and that the tumor diameter of HpUIGC was significantly smaller than that of HpGC [21]. The results of this study are similar to those in previous reports, with the mean age of patients with early HpUIGC being 55.7 ± 10.9 years and a median tumor size of 5 mm, which are considered important characteristics for detecting early HpUIGC. In the present study, the locations of the lesions were plotted with reference to Yoshimura et al. [22]. The most common SRCC cases in the present study were all located in the border region between the fundus and pyloric glands, similar to previous reports. Furthermore, no 0-I or 0-IIa lesions were found in the SRCC. Therefore, when observing the border region in the *H. pylori*-uninfected stomach, we should note the small fading 0-IIb and 0-IIc lesions. The next most common type was GA-FG, characterized by 72.7% of cases being located in the upper area, and 72.7% of cases were type 0-IIa. Consequently, when observing the upper area in the *H. pylori*-uninfected stomach, small 0-IIa lesions should be noted. Additionally, 81.8% of GA-FG cases were at a depth of SM1. Although GA-FG easily invades the SM, it may not be highly malignant [23], and the results of future studies are awaited. In the present study, SRCC and GA-FG accounted for 3/4 of the total histological types. Conversely, 16.7% of the cases in the present study had GA-FV-RS. Recently, GA-FV, diagnosed as a differentiated type of GC with low-grade atypia in HpUIGC, has been classified as a subtype of raspberry-shaped GC cases. These cancers exhibit reddish, protruding lesions with a finely granular surface [24,25,26]. The World Health Organization considers GA-FV as a foveolar-type adenoma; contrarily, according to the JCGC, GA-FV is a well-differentiated adenocarcinoma [24,25,27]. In the present study, GA-FV-RS was located in the greater curvature in all cases. Suzuki et al. also reported that 97.7% of cases of the foveolar type were located in the greater curvature, which may be one of the characteristics of GA-FV-RS [25]. Furthermore, 83.3% of GA-FV-RS had 0-I lesions in this study, which may also be a distinctive characteristic. All patients were able to undergo en bloc resection; however, one case of GA-FG showed a positive result for the vertical margin. Because most GA-FG cases are at SM1 depth, careful consideration of the depth at which the lesion is dissected is necessary when performing ESD.

The long-term prognosis of early HpUIGC is not yet clear. Although the number of cases in this study was very small, the 3-year prognosis was good. Regarding biological behavior, GA-FG is considered less aggressive because it exhibits low cellular atypia, no vascular invasion, low proliferative activity, a lack of p53 protein overexpression, and a good prognosis [28]. In addition, the proliferative potential of cancer cells tends to be lower in undifferentiated HpUIGC cases than in *H. pylori*-positive cases [29]. However, there have been reports of HpUIGC in advanced stages, and we believe that this is a cancer that should not be overlooked [30,31].

The present study had several limitations. First, our sample size was relatively small. Second, data on advanced GC were not collected. Although the early stage of HpUIGC was the focus of this study, advanced cancers should also be considered to determine the current status of all HpUIGC cases. Third, we were unable to investigate in detail the pathology of GCs other than SRCC, GA-FG, and GA-FV-RS. All other cancers were of the differentiated type; however, no complete gastric type was observed. Epstein–Barr virus infection [32], autoimmune gastritis [33], germline mutations in the *CDH1* gene [34], polymorphism in the prostate stem cell antigen [35], and familial adenomatous polyposis [36] are known to be associated with HpUIGC. Furthermore, there are reports of differentiated carcinomas with a gastrointestinal phenotype found in the antrum [21]. Fourth, in the present study, gastric adenocarcinoma of fundic-gland mucosa-type (GA-FGM) was also considered as GA-FG. GA-FGM is a more aggressive tumor than GA-FG and requires caution [37]. Fifth, GA-FV of whitish flat-elevated appearance, which has been reported as a foveolar-type [21], was not observed in this study. Sixth, although not observed in the Kagoshima Prefecture data, the decline in the number of cases in 2020 and 2021 in the NDB data may have been influenced by the COVID-19 pandemic. There have been reports of a decrease in the diagnosis and treatment of curable early-stage GC, as well as a decrease in the number of surgeries, following the COVID-19 pandemic [38,39,40]. While this observation in the NDB data does not affect the primary endpoint of the current study, further verification of the discrepancy with the data from Kagoshima Prefecture is needed. Seventh, this study included data from Kagoshima Prefecture and not from all regional cities in Japan where the population is aging. Further research is needed to determine if regional cities outside of Kagoshima Prefecture are experiencing similar trends. Eighth, this study was unable to compare the clinical features, outcomes of ESD, and prognosis of ESD in HpUIGC and HpGC. Comparing these data is crucial for a deeper understanding of HpUIGC. Studies comparing cases of undifferentiated types based on *H. pylori* infection status found that *H. pylori*-uninfected patients were significantly more likely to be male and younger and to have tumors located in the lower third of the stomach, more discolored dominant lesions, smaller lesions, and pure SRCCs [41]. However, the primary endpoint of this study was to determine the trend in the proportion of HpUIGC among EGCs treated with ESD in an aging population in Japan. No data were collected on the endoscopic characteristics of HpGC, ESD outcomes, or prognosis. Further research is needed on these aspects. Ninth, in Kagoshima Prefecture, population-based GC screening began in 2023, and there were no changes in the health screening process during the period studied. However, improvements in endoscopic techniques and the spread of knowledge about HpUIGC may have contributed to an increase in the detection rate of HpUIGC.

## 5. Conclusions

The proportion of ERs for early HpUIGC, which are more common in relatively young patients, may be increasing as a proportion of all ERs for GC, even in areas of Japan with an aging population. Therefore, it is crucial for gastroenterologists to comprehend the current status of early HpUIGC in regional areas.

## Figures and Tables

**Table 1:** Characteristics of the *Helicobacter pylori*-uninfected gastric cancer patients and lesions.

	Values
**Patients, n**	35
**Age, mean ± SD (years)**	55.7 ± 10.9
**Sex, n (%)**	
Men	26 (74.3)
Women	9 (25.7)
**Opportunity to detect lesions, n (%)**	
Checkups or periodic EGD	34 (97.1)
Symptomatic	0 (0)
Other	1 (2.9)
**Resection modality, n (%)**	
ESD	31 (88.6)
EMR	4 (11.4)
**Tumor location, n (%)**	
U	14 (40.0)
M	10 (28.6)
L	11 (31.4)
Ant	5 (14.3)
Post	4 (11.4)
Less	5 (14.3)
Gre	21 (60.0)
**Macroscopic type, n (%)**	
0-I	6 (17.1)
0-IIa	14 (40.0)
0-IIb	8 (22.9)
0-IIc	7 (20.0)
**Lesion size, median (mm)**	5 [0.5–72]
**Histological type, n (%)**	
SRCC	14 (40.0)
GA-FG	11 (31.4)
GA-FV-RS	6 (17.1)
Other type	4 (11.4)
**Lesion depth, n (%)**	
Mucosa	23 (65.7)
Submucosa 1	10 (28.6)
Submucosa 2	2 (5.7)
**Positive for lymphatic invasion, n (%)**	1 (2.7)
**Positive for venous invasion, n (%)**	1 (2.7)

SD, standard deviation; EGD, esophagogastroduodenoscopy; ESD, endoscopic dissection; EMR, endoscopic mucosal resection; U, upper third of stomach; M, middle third of stomach; L, lower third of stomach; Ant, anterior, Post, posterior, Less, lesser curvature, Gre, greater curvature; SRCC, signet-ring cell carcinoma; GA-FG, gastric adenocarcinoma of the fundic-gland-type; GA-FV-RS, foveolar-type adenocarcinoma with a raspberry shape.

**Table 2:** Treatment outcome.

	Values
**En bloc resection rate, n (%)**	35 (100)
**Horizontal margin positive rate, n (%)**	0 (-)
**Vertical margin positive rate, n (%)**	1 (2.9)
**Evaluation of curability, n (%)**	
eCura A	24 (68.6)
eCura B	8 (22.9)
eCura C	3 (8.6)
**Complications, n (%)**	
Intraprocedural perforation	1 (2.9)
Delayed perforation	0 (-)
Delayed bleeding	0 (-)

**Table 3:** Clinicopathological features.

	SRCC (*n* = 14)	GA-FG (*n* = 11)	GA-FV-RS (*n* = 6)	Other Type (*n* = 4)
**Age, mean ± SD (years)**	50.1 ± 10.1	62.5 ± 8.0	51.0 ± 6.3	63.5 ± 13.1
**Macroscopic type, n (%)**				
0-I	0 (-)	1 (9.1)	5 (83.3)	0 (-)
0-IIa	0 (-)	8 (72.7)	1 (16.7)	4 (100)
0-IIb	8 (57.1)	0 (-)	0 (-)	0 (-)
0-IIc	6 (42.9)	2 (18.2)	0 (-)	0 (-)
**Lesion size, median (mm)**	5.0 [0.5–10]	6.0 [3.0–13]	3.5 [3.0–5.0]	16.5 [11–72]
**Lesion depth, n (%)**				
Mucosa	12 (85.7)	2 (18.2)	6 (100)	3 (75.0)
Submucosa 1	1 (7.1)	9 (81.8)	0 (-)	0 (-)
Submucosa 2	1 (7.1)	0 (-)	0 (-)	1 (25.0)

SD, standard deviation; SRCC, signet ring cell carcinoma; GA-FG, gastric adenocarcinoma of the fundic-gland-type; GA-FV-RS, foveolar-type adenocarcinoma with a raspberry shape.

**Table 4:** Change of the proportion of *Helicobacter pylori*-uninfected gastric cancer to the total number of endoscopic resections for early gastric cancer.

	2010–2015	2016–2021	*p* Value
**Hp-uninfected,** **n (%)**	5 (0.7)	25 (2.1)	0.02
**Hp current/previous infection, n (%)**	683 (99.3)	1172 (97.9)	

Hp, *Helicobacter pylori*.

## Data Availability

Data are contained within the article.
